# The Finding My Way UK Clinical Trial: Adaptation Report and Protocol for a Replication Randomized Controlled Efficacy Trial of a Web-Based Psychological Program to Support Cancer Survivors

**DOI:** 10.2196/31976

**Published:** 2021-09-20

**Authors:** Nicholas J Hulbert-Williams, Monica Leslie, Lee Hulbert-Williams, Bogda Koczwara, Eila K Watson, Peter S Hall, Laura Ashley, Neil S Coulson, Richard Jackson, Sue Millington, The Finding My Way UK Trial Steering Group, Lisa Beatty

**Affiliations:** 1 Centre for Contextual Behavioural Science School of Psychology University of Chester Chester United Kingdom; 2 College of Medicine and Public Health Flinders University Adelaide Australia; 3 Department of Medical Oncology Flinders Medical Centre Adelaide Australia; 4 Oxford Institute of Nursing, Midwifery & Allied Health Research Oxford Brookes University Oxford United Kingdom; 5 Edinburgh Cancer Research Centre University of Edinburgh Edinburgh United Kingdom; 6 Leeds School of Social Sciences Leeds Beckett University Leeds United Kingdom; 7 School of Medicine University of Nottingham Nottingham United Kingdom; 8 Liverpool Clinical Trials Centre University of Liverpool Liverpool United Kingdom; 9 Cancer Survivor Research Partner Chester United Kingdom; 10 see Authors’ Contributions; 11 College of Education Psychology & Social Work Flinders University Adelaide Australia

**Keywords:** cancer, survivorship, psychosocial intervention, digital health, quality of life, protocol, mobile phone

## Abstract

**Background:**

Cancer survivors frequently report a range of unmet psychological and supportive care needs; these often continue after treatment has finished and are predictive of psychological distress and poor health-related quality of life. Web-based interventions demonstrate good efficacy in addressing these concerns and are more accessible than face-to-face interventions. *Finding My Way* (FMW) is a web-based, psycho-educational, and cognitive behavioral therapy intervention for cancer survivors developed in Australia. Previous trials have demonstrated that *FMW* is acceptable, highly adhered to, and effective in reducing the impact of distress on quality of life while leading to cost savings through health resource use reduction.

**Objective:**

This study aims to adapt the Australian *FMW* website for a UK cancer care context and then undertake a single-blinded, randomized controlled trial of *FMW UK* against a treatment-as-usual waitlist control.

**Methods:**

To an extent, our trial design replicates the existing Australian randomized controlled trial of *FMW.* Following a comprehensive adaptation of the web resource, we will recruit 294 participants (147 per study arm) from across clinical sites in North West England and North Wales. Participants will have been diagnosed with cancer of any type in the last 6 months, have received anticancer treatment with curative intent, be aged ≥16 years, be proficient in English, and have access to the internet and an active email address. Participants will be identified and recruited through the National Institute for Health Research clinical research network. Measures of distress, quality of life, and health economic outcomes will be collected using a self-report web-based questionnaire at baseline, midtreatment, posttreatment, and both 3- and 6-month follow-up. Quantitative data will be analyzed using intention-to-treat mixed model repeated measures analysis. Embedded semistructured qualitative interviews will probe engagement with, and experiences of using, *FMW UK* and suggestions for future improvements*.*

**Results:**

The website adaptation work was completed in January 2021. A panel of cancer survivors and health care professionals provided feedback on the test version of *FMW UK.* Feedback was positive overall, although minor updates were made to website navigation, inclusivity, terminology, and the wording of the *Improving Communication* and *Sexuality and Intimacy* content. Recruitment for the clinical trial commenced in April 2021. We aim to report on findings from mid-2023.

**Conclusions:**

Replication studies are an important aspect of the scientific process, particularly in psychological and clinical trial literature, especially in different geographical settings. Before replicating the *FMW* trial in the UK setting, content updating was required. If *FMW UK* now replicates Australian findings, we will have identified a novel and cost-effective method of psychosocial care delivery for cancer survivors in the United Kingdom.

**Trial Registration:**

International Standard Randomized Controlled Trial Number (ISRCTN) 14317248; https://www.isrctn.com/ISRCTN14317248

**International Registered Report Identifier (IRRID):**

DERR1-10.2196/31976

## Introduction

### Background

Cancer survivorship rates in the United Kingdom have increased such that up to 57% of cancer patients in the United Kingdom can now expect to survive for 10 years [1]. However, there is regional discrepancy, and survival rates differ across geographic regions and treatment center catchment areas. Recently published screening studies suggest a prevalence of distress of up to 41.5% in adolescents and young adults [2] and 46% in adult [3] cancer populations. Anxiety, depression, and other psychological comorbidities significantly impact quality of life [4]. If left untreated, distress can escalate [5], and a pooled analysis of 163,363 cancer survivors demonstrated that distress in some cancer groups predicted higher mortality risk, even after controlling for age, sex, education, socioeconomic status, BMI, smoking, and alcohol intake [6]. Our own work in people diagnosed with the four most common cancers demonstrates that cognitive and emotional responses to diagnosis can predict distress [4] and that psychological variables, such as psychological flexibility, are predictive of distress-related outcomes *independent* of clinical and sociodemographic characteristics [7]. Unmet psychological and supportive care needs are prevalent in cancer survivors [8-11]; we found that 54% of hematological patients report five or more unmet supportive care needs [10], and 46% of colorectal patients report at least one specific psychological need [12]. Therefore, there is a crucial need to develop effective interventions to manage psychological distress in cancer survivors.

A recent review of psychological interventions for patients with cancer [13] concluded that although cognitive behavioral therapy remains the gold standard treatment choice, we need more methodologically robust research to determine efficacy and scope for implementation. There is an excess of small-scale studies where fully powered trials exploring moderators and mediators of effects are needed. Research in non–breast cancer populations is recommended, along with the inclusion of health economic outcomes, to provide powerful data for clinical service commissioners. Given the high cost and time invested in developing new intervention content and delivery formats, one effective strategy is to adapt *existing* interventions rather than waste finite resources to develop novel and competing interventions. Furthermore, replication studies are important to demonstrate consistency and generalizability of outcomes and are recommended in response to the replication crisis in psychology [14].

A recent systematic review highlighted that one of the top barriers to accessing psychosocial support identified by patients with cancer is difficulty with transport to the health service delivery center [15], although other types of access issues have been reported elsewhere. To overcome this barrier, recent research has increasingly investigated the feasibility and efficacy of web-based psychosocial interventions for patients with cancer [16]. Web-based delivery methods are also recommended to overcome the expense of delivering psychological support [17-19]. Given the recent increases in home-based internet access in the United Kingdom, especially through the rapid development and uptake of smartphone and tablet technologies [20], web-based interventions may address access issues by widening the potential pool of beneficiaries [21]. They also overcome the stigma associated with overtly seeking psychological support [22], and (as demonstrated through the current COVID-19 pandemic) are a way to ensure continuation of service where there may be barriers to continued face-to-face care [23]. Digital psychosocial interventions confer many potential benefits, including greater convenience, reduced burden on patients with cancer and caregivers, and reduced resource use and health care costs, as compared with traditional face-to-face interventions [24]. However, most clinically measurable differences associated with web-based psychosocial interventions for this population fail to meet statistical significance, a phenomenon likely attributable to study design rather than a lack of real effect [16]. In addition, there is a need to identify treatment components involving active user engagement with web-based exercises to mitigate the lack of face-to-face interaction.

One of the most promising web-based interventions for cancer survivors is *Finding My Way* (FMW), developed by Beatty et al [25] in Australia. FMW is the second iteration of a six-module, web-based, self-guided intervention, initially titled *Cancer Coping Online* [26]. It uses psycho-educational and cognitive behavioral therapy–based theoretical frameworks and includes exercises from third-wave approaches, for example, mindfulness and values clarification work exercises. Early pilot work demonstrated benefits for physical functioning and distress outcomes [18]. Although between-group differences were not replicated in a recent, larger, randomized controlled trial (RCT) [27], this trial compared the intervention group with a low-dose active control group (identical psychoeducation and video-based content), with both groups reporting reductions in distress over time. As such, the lack of significant between-group findings may be related more to the overlap of content between treatment groups rather than a lack of efficacy in the web-based intervention group. This recent RCT found significantly better emotional functioning and lowered health care use in the FMW arm, demonstrating both (1) that distress had less functional impact on quality of life and (2) health service cost reduction [27]. Adherence was also high [26]. A number of replication studies of FMW are underway across the world, and FMW has been adapted for women with advanced breast cancer [28], demonstrating the flexibility of the program for different demographic groups and clinical contexts. As such, FMW is a good candidate for effective support in the UK cancer care setting, but some adaptation was necessary before implementation.

### Objective

This paper reports on our work undertaken to adapt the intervention, and the protocol for the ongoing RCT, which tests its efficacy in a UK National Health Service (NHS) setting. We aim to test (1) whether outcome effects are replicated or improved and (2) whether intervention uptake, use, and acceptability meet feasibility thresholds for implementation in standard care.

## Methods

### Trial Design

We will conduct a single-blinded RCT of FMW UK compared with treatment-as-usual control. Mixed methods data collection—using self-report questionnaires, quantitative clinical data extraction, and in-depth interviews—will be undertaken to investigate efficacy and acceptability. Where possible, trial design and outcomes replicate the key features of the Australian RCT of FMW by Beatty et al [27].

All aspects of study design and governance are planned to involve the expert voices of people affected by cancer as active partners in the research study. The University of Chester hosted the study with scrutiny provided by a trial steering group comprising grant coapplicants (including a cancer survivor coapplicant), the local research team, a patient, a caregiver, and a health care professional stakeholder representative. The steering group meets twice per year, with a smaller project management group meeting bimonthly to provide operational oversight. The funder peer-review report is presented in Multimedia Appendix 1.

### The FMW UK Intervention

The FMW UK intervention is designed as a six-week, self-administered, modularized, web-based program. Written and video-based information about a range of cancer care topics and the provision of psychological intervention materials are supplemented with testimonials from cancer survivors sharing their experiences and advice. Interactive exercises, including worksheets, assessment tools, and prerecorded self-guided mindfulness meditations are included; these experiential components are likely to boost efficacy [27]. The modules, released one per week, address common psychosocial concerns and unmet needs among cancer survivors and are structured around (1) treatment and communication with treatment teams; (2) coping with physical symptoms and side effects; (3) managing distress; (4) challenges to identity, body image, and sexuality; (5) social support and family concerns; and (6) issues that arise after treatment. On first accessing the site, users are prompted to choose the order in which they wish to access modules to meet their self-determined need priorities. A booster module is released one month after completion, which recaps program content and signposts back to earlier modules.

### Contextual Adaptation

We began our adaptation of FMW with our local research team reviewing the information provided to determine which aspects needed to change for the UK cancer care setting. This included referencing standard care pathways and services available to patients with cancer in the United Kingdom and adapting some terminology to avoid confusion. We reviewed all website content to identify Australian-specific resources and treatment information and then worked with our steering group (including academics, clinicians, and patient and caregiver representatives) to systematically identify equivalent British information and signposting resources with which to replace them. Our adaptation plan was approved by the trial steering group.

### Video Content

Each module included an information video, and in the Australian version, these were recorded by either an oncologist or a psychologist. In rerecording these videos, we chose to include a wider variety of professionals, including psychologists, oncologists, surgeons, and managers of local cancer support centers with a cancer-nursing background. This change was undertaken to (1) better represent the multidisciplinary nature of cancer care in the UK setting and (2) as a tool to increase diversity and inclusivity throughout the program. These videos were scripted, including only minor edits from the original Australian content.

Although much of the content of the cancer survivor testimonial videos was applicable to a UK-based cohort, we produced a new set of videos with cancer survivors from the United Kingdom to maximize the extent to which our participants would connect and affiliate with the stories and experiences shared. Using our existing networks, advocacy groups, and advertisements placed on social media, we recruited nine cancer survivors from across North Wales and the North West of England (Table 1) and undertook individual video-recorded interviews with each, between August and September 2020. Survivors were selected to maximize the diversity of interviews, both demographically and with regard to cancer experiences. Video interviews were unscripted but followed a standard question schedule (Textbox 1) that had been used in the development of the original Australian website and that was provided to interviewees in advance for preparation purposes. Videos were reviewed by three team members to select clips that were edited into thematically linked videos for each module.

**Table 1:** Characteristics of the cancer survivors who participated in video interviews for the Finding My Way UK website.

Name^a^	Gender	Age (years)	Cancer type	Time from diagnosis (years)
Janet	Female	64	Bowel	3
Martin	Male	66	Prostate	>10
Sue M	Female	56	Breast	6
Dylan	Male	47	Bladder	2
Terry	Male	74	Lung	>10
Sue H	Female	61	Breast	4
Bernadette	Female	52	Breast	1
Sophie	Female	24	Burkitt lymphoma	4
Babz	Male	31	Non-Hodgkin lymphoma	>10

^a^Participants were given the choice to use their actual names or pseudonyms.

**Question schedule for video interviews with cancer survivors to create the Finding My Way UK intervention content.**

Question schedule

1. What issues came up for you after diagnosis (and during treatment) in terms of making decisions about treatment, or when discussing things with your medical treatment team?
2. During treatment, what was your most pressing physical need/concern?
3. During treatment, what was your most pressing emotional need/concern?
4. Some people find that many of their roles change during treatment, and that they aren’t able to do the tasks and activities they usually do, which then affects the way they feel about themselves. During treatment, how did your roles change and how did this affect you?
5. During treatment, what was your most pressing social need? What surprised you?
6. What things were challenging for you with your family life?
7. If you could give one piece of advice to another person with cancer, what would it be?
8. Over the process of treatment, what was the most confusing issue for you?
9. What did you do to mark the end of your treatment?
10. What advice would you give to other cancer survivors about staying healthy?
11. Some people say that having cancer gave them an opportunity to learn something new about life or themselves. What is the one learning experience you had that you would not have had if you did not have cancer?
12. Were there any other questions you thought we should have asked?

Video interview participants (survivors and health care professionals) were reimbursed for their time and travel expenses, as is good practice for patient and public involvement in health research [29]. All participants signed a consent form to permit the ongoing use of their video content after the trial was complete. Given that these interviews took place during the COVID-19 pandemic, a rigorous health and safety assessment was undertaken, and appropriate infection control measures were implemented. Video recordings (and later editing work) were undertaken by the research team, given the difficulties inherent in commissioning this work to an external company through the intermittent implementation of COVID-19 related social distancing in the United Kingdom during this time.

### Evidence Review

Given that the Australian FMW content was last updated in 2013, we reviewed all research claims made throughout the website content and conducted literature reviews to identify which claims were still upheld by recent research. We subsequently updated the references for some evidence statements and edited claims that were no longer conclusively supported by the current evidence base. In brief, this includes the following:

- De-emphasizing the strength of claims made about the benefits of emotional expression and therapeutic writing [30,31].
- Updated references in relation to benefit finding and positive adjustment [7].
- Updated reference to support our recommendation for the benefits of mindfulness-based exercises [32].
- Inclusion of more recent references in relation to the impact of dyadic influences on adjustment between patients and their partners [33] and in relation to the benefits of information on distress levels in close others of people being treated for cancer [34].
- Reframing of claims made about the benefits of religious and spiritual beliefs to confirm that these may be helpful for those with existing beliefs, but that we are not seen as advocating a change in practices or beliefs.

### Web Hosting and User Testing

We commissioned an independent web design company to adapt the original FMW web-based framework for our purposes. The website was designed using *Wordpress v.5.7.1* (WordPress Foundation) and was hosted through *Kinsta.* Videos are uploaded to *YouTube* with embedded links provided at relevant points on the website. The videos are not publicly listed to prevent access outside of the trial, and the FMW UK website is restricted to only those with a username and password provided by our team.

Once an initial test website had been created, we recruited a panel of four cancer survivors and three health care professionals to provide user feedback, each of whom was financially compensated for their input. Cancer survivors were identified from our initial advertisement for video interview participants, and health care professionals (oncology and psychology-based) were identified from existing professional networks. Additional user testing was performed by the trial steering group. Where relevant, feedback was integrated into a final website update (see the *Results* section) before recruitment commencing.

### Participants

#### Sample Size Calculation

Calculations were based on the primary outcome of change in cancer-specific distress between the two patient groups. The original FMW RCT sample size calculation [27] used a standardized effect size of 0.35 and an SD of 4 units, which equates to an absolute change in cancer distress scores of 1.4 units. This study observed a larger than expected SD and we propose a sample size based on a conservative estimate of the residual SD of 7 units accordingly (but keeping the clinically relevant difference at the aforementioned 1.4 units). The correlation between successive measurements on the same patient is assumed to be high, and so a conservative *r*=0.70 was used. Sample size calculations were performed assuming a paired two-tailed *t* test using the derived SD of the change in the primary outcome of 5.42. Assuming a patient attrition of 20% and α of .05, 294 patients (147 per study arm) are required for a statistical power of 80% [35]. We will allow up to 30% overrecruitment to mitigate the effects of missing data and to allow for at least minimal recruitment of less common cancer types.

#### Recruitment and Eligibility Criteria

Participants will be recruited from multiple NHS hospital sites across North West England and North Wales using the National Institute for Health Research (NIHR) Clinical Research Network (CRN) research nurses (RNs). Patients will be eligible to take part if they meet the following inclusion criteria: (a) have been diagnosed with cancer of any type in the past six months, (b) received anticancer treatment with curative intent, (c) are aged 16 years or older, (d) are sufficiently proficient in English to provide informed consent and use the program; and (e) able to access the internet and have (or be willing to set up) an email address. Patients will be ineligible or excluded if they have a severe comorbidity considered to interfere with the individual’s ability to complete the requirements of the study or to provide informed consent (eg, intellectual disability or neurological impairment). Nurses will complete fortnightly screening logs to provide anonymized information on the number of patients screened, eligible, and then provided with a trial information pack to inform later potential implementation decisions.

CRN RNs will screen regular multidisciplinary team meeting records for eligible patients and identify when their next clinical appointment will be. The CRN RN will approach each patient face-to-face to tell them about the study and provide an information pack. Where no appointment is planned within the subsequent 6 weeks, or where face-to-face introduction would be otherwise problematic (eg, lack of private space to discuss the study), our protocol permits a telephone introduction to the study. At the start of recruitment, records of existing multidisciplinary team meetings will be retrospectively searched for any patients meeting the eligibility criteria, although we anticipate that the majority of our sample will be recruited through prospective recruitment over a 12-month recruitment period.

Assuming a conservative 40% consent rate [27], we estimate that 735 patients will need to be approached to reach our target sample size. We will recruit a range of cancer teams to ensure clinical diagnostic and demographic variability.

### Procedure

**Figure 1:**
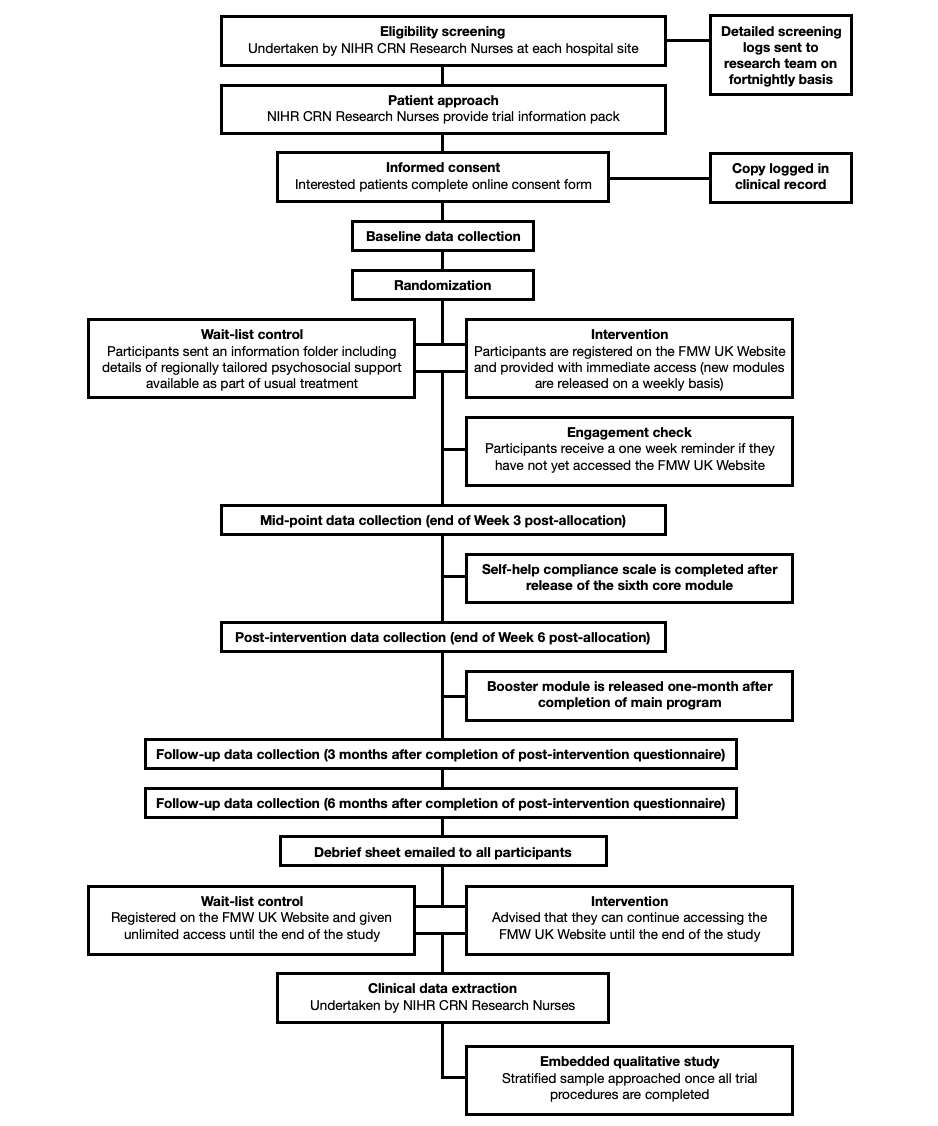
Procedure for the Finding My Way UK Clinical Trial. CRN: Clinical Research Network; FMW: Finding My Way; NIHR: National Institute for Health Research.

After reading information provided by the CRN RNs, patients wishing to take part in the study can access our study recruitment website via a link in their information pack. This provides a full trial information sheet and access to a web-based consent form. Once their consent is submitted, participants are redirected immediately to the baseline survey via the *Qualtrics survey platform* (Qualtrics). Participants also will receive an automated email with a link to complete the baseline questionnaire at a later date or in a number of sittings if they prefer*.* Upon full completion of the baseline questionnaire, an unblinded member of the research team will complete the study arm allocation using a computerized randomization allocation system using *REDCap* software (Vanderbilt University) [36]. The randomization algorithm was set up to ensure equal numbers of participants in both the intervention and control arms, stratified by cancer diagnosis to ensure the spread of patients across both trial arms. The randomization system was set up and is overseen by the Liverpool Clinical Trials Centre. Following allocation, participants are either emailed account details to access the FMW UK website (intervention group) or sent a PDF copy of a site-specific information pack listing existing local and national sources of psychosocial support that they can access as part of treatment as usual (control group; Figure 1). Control participants also have the option to receive a hard copy of the information pack via the post.

Those given immediate access to FMW UK are encouraged to log in within one week, study the instruction materials provided, and select the order in which they would like to receive access to the intervention modules (if no preference is given, participants receive in default numerical order). A reminder (text or phone, as preferred) is then sent if they have not logged in during this time. Modules are then automatically released once per week; access to the booster module is also released one month after completion of the main program. Regular automated email reminders are sent as new modules are released each week.

At the point of being informed of intervention allocation, automated email reminders to complete the study questionnaires are set up through *Qualtrics*. Text messages or phone call reminders (as preferred) are sent for a period of 7 days without submission of any specific questionnaire. Questionnaires are completed at the end of the third (midintervention) and sixth (posttreatment) week, and then at 3- and 6-month follow-ups, both timed from the release of the posttreatment questionnaire. Participants are sent a debrief sheet at this point, and control arm participants are granted access to the FMW UK website. At this point, CRN RNs complete clinical data extraction from hospital records using a standard form; this includes information about the date of diagnosis, primary or recurrent diagnosis, curative or palliative treatment intent, principle treatment approach adopted (surgery, chemotherapy, radiotherapy, or watch-and-wait), date of the end of active treatment (if applicable), date of any recurrence or relapse (if applicable), date of death (if applicable), known referrals to mental health care teams since diagnosis, number of days of inpatient care since study enrollment and types of health care professionals seen during these stays, the number of outpatient visits since study enrollment and types of health care professionals seen during these visits, and any diagnostic tests conducted since study enrollment. As this study is registered on the UK NIHR CRN Portfolio, costs for most CRN RN activities (both recruitment and clinical data collection) are covered by CRN Study Support Services, with costs for additional archiving at each site reimbursed by the clinical trial research grant.

### Measures

#### Study Outcomes

We will ask participants to self-report the following demographic characteristics: age, gender, sexuality, ethnicity, employment, education, marital status, household income, and postcode (to calculate the index of multiple deprivation). The following list of self-report questionnaires is then administered throughout the study (Table 2). We sought to use measures consistent with the original Australian FMW study to most closely replicate this previous clinical trial. Exceptions include (1) a briefer measure was identified to reduce participant burden (eg, using the Psychological Impact of Cancer Scale [37] rather than the mini-Mental Adjustment to Cancer Scale [38]); (2) an additional measure was required to assess psychological flexibility, our hypothesized mediator of the intervention effect; and (3) a UK-specific measure of health resource use was needed for context-specific health economic assessment.

**Table 2:** Schedule of questionnaire administration.

Variable	Baseline assessment	Midpoint assessment	Beginning of sixth module	End of Finding My Way UK	3-month follow-up	6-month follow-up
Demographic characteristics	✓^a^					
Cancer-specific distress (Post-Traumatic Stress Scale)	✓	✓		✓	✓	✓
Psychological well-being (Depression, Anxiety, and Stress Scales 21-item version)	✓	✓		✓	✓	✓
Quality of life (QLQ-C30^b^)	✓	✓		✓	✓	✓
Psychological adjustment to cancer (the Psychological Impact of Cancer Scale)	✓	✓		✓	✓	✓
Health care use (the UK Cancer Costs Questionnaire)	✓	✓		✓	✓	✓
Perceived social support (the Medical Outcome Study Social Support Survey)	✓					
Emotion regulation (Difficulties in Emotion Regulation Scale)	✓					
Information-seeking preferences (the Miller Behavioral Style Scale)	✓					
Psychological flexibility (the CompACT Questionnaire)	✓	✓		✓	✓	✓
Engagement with intervention (Self-Help Compliance Scale)			✓			

^a^Assessment performed.

^b^QLQ-C30: European Organisation for Research and Treatment of Cancer Quality of Life Core Questionnaire.

#### Primary Outcome

The primary trial outcome variable was cancer-specific distress*.* For this variable, we will use the Post-Traumatic Stress Scale [39], a 17-item measure in which participants respond on a 4-point Likert scale, where responses are anchored from 0 (*not at all or only one time*) to 3 (*5 or more times per week or almost always*). The Post-Traumatic Stress Scale is associated with excellent internal consistency reliability (α=.91) [39] and has good concurrent validity, including strong positive correlations with other measures of trauma-related intrusion and avoidance, anxiety, and depression [39]. Higher scores on the Post-Traumatic Stress Scale indicate a greater severity of cancer-specific distress.

#### Secondary Outcomes

##### Psychological Well-being

The Depression, Anxiety, and Stress Scales, 21-item version [40] is a short measure of negative emotions experienced over the course of the past week for the individual. Each item is scored on a 4-point Likert scale, anchored from 0 (*did not apply to me at all*) to 3 (*applied to me very much or most of the time*). Total scores for each subscale of the Depression, Anxiety, and Stress Scales, 21-item version can be calculated, where higher scores indicate greater levels of depression, anxiety, and stress. The scale has good internal reliability (depression, α=.91; anxiety, α=.81; stress, α=.89), and concurrent validity, including strong positive correlations with other measures of depressive symptoms and anxiety [40].

##### Quality of Life

The European Organisation for Research and Treatment of Cancer Quality of Life Core Questionnaire (QLQ-C30) [41] is a 30-item quality of life assessment for cancer patients, which yields a global quality of life score and five functional subscale scores associated with physical, emotional, social, role, and cognitive quality of life domains. In total, 28 items are presented on a 4-point Likert scale ranging from 1 (*not at all*) to 4 (*very much*). The final two items assessing subjective assessment of overall health and quality of life are presented on a 7-point Likert scale anchored from 1 (*very poor*) to 7 (*excellent*). The global score for the QLQ-C30 is associated with good internal consistency reliability (α=.86) and has good concurrent validity, with both global quality of life and domain subscales significantly positively correlated with performance status throughout treatment [41]. A higher global score on the QLQ-C30 indicates a greater quality of life. The QLQ-C30 score can be converted into an indication of Quality-Adjusted Life Years for use in health economic analysis.

##### Psychological Impact of Cancer

The Psychological Impact of Cancer Scale [37] is a 12-item self-report measure of psychological adjustment to cancer. Each item is presented on a 4-point Likert scale anchored from 1 (*definitely does not apply to me*) to 4 (*definitely applied to me*). The Psychological Impact of Cancer Scale yields four subscale scores: cognitive distress, cognitive avoidance, emotional distress, and fighting spirit. Greater scores on each subscale indicate greater levels of the named construct (eg, a greater score on the Cognitive Distress subscale indicates greater levels of cognitive distress). The fighting spirit subscale will not be included because of underlying psychometric property issues [37]; the remaining three scales have reasonable internal consistency reliability (α≥.62) and good concurrent validity with longer measures of psychological adjustment to cancer [37].

##### The UK Cancer Costs Questionnaire

The UK Cancer Costs Questionnaire [42] is a flexible modular self-report measure of resource use by people with cancer and those with a previous diagnosis of cancer. The UK Cancer Costs Questionnaire assesses employment status, family support provided, government benefits received, and support provided by other organizations over the previous 3 months. The UK Cancer Costs Questionnaire prioritizes brevity to minimize the burden of data collection for participants. For full health care use outcome data, this self-report questionnaire is supplemented by health service resource use data extracted from clinical records and the calculation of Quality-Adjusted Life Years from the QLQ-C30.

#### Potential Intervention Moderator or Mediators

##### Rationale for Moderator and Mediator Analyses

In psychological intervention research, it is important to include measures of the hypothesized variables being acted upon to (1) verify cause-and-effect relationships on outcome improvements and (2) identify any important moderator and mediator analyses that may need to be undertaken [13]. The following measures were identified as likely moderators of the effectiveness of the intervention and have been informed in large part by a moderator analysis of the Australian FMW Trial [43].

##### Perceived Social Support

The Medical Outcome Study (MOS) Social Support survey [44] is a 20-item measure, with items presented as a 5-point Likert scale anchored from 1 (*none of the time*) to 5 (*all of the time*). The MOS Social Support Survey yields four subscale scores: emotional or informational support, tangible support, affectionate support, and positive social interactions. Each subscale is associated with excellent internal consistency reliability (α>.91) [44]. The MOS Social Support Survey is associated with good convergent validity with measures of family ties, family functioning, and mental health, and good divergent validity with measures of purely physical health [44]. Higher scores on individual subscales and the overall support index indicate greater social support.

##### Emotion Regulation

The Difficulties in Emotion Regulation Scale [45] is a 36-item self-report measure of six dimensions of emotion regulation difficulties: lack of awareness of emotional responses, lack of clarity of emotional responses, nonacceptance of emotional responses, limited access to emotion regulation strategies perceived as effective, difficulties controlling impulses when experiencing negative emotions, and difficulties engaging in goal-directed behaviors when experiencing negative emotions. Each item is scored on a 5-point Likert scale ranging from 1 (*almost never*) to 5 (*almost always*). The global difficulties in emotional regulation scale is associated with excellent internal consistency reliability (α=.93), and each subscale is associated with good internal consistency reliability (α>.80) [45]. The Difficulties in Emotion Regulation Scale is associated with good construct validity and predictive validity [45]. Higher scores on the Difficulties in Emotion Regulation Scale indicate greater problems with emotion regulation.

##### Information-Seeking Preferences

The Miller Behavioral Style Scale [46] is a self-report measure of information-seeking preferences. The scale identifies individual preferences for seeking threat-related cues (monitors) versus seeking distraction to minimize exposure to threat-related cues (blunters). The scale prompts participants to imagine four stressful scenarios, each of which is followed by eight statements that describe different ways of coping with the stressor. Participants are asked to select all the statements that apply to them. The Miller Behavioral Style Scale is associated with good test-retest reliability over a 4-month period (monitoring subscale *r*=0.72; blunting subscale *r*=0.75) and high construct validity, as indicated by high correspondence with information-seeking behavior in a stress-inducing laboratory task [46]. Higher scores on the Miller Behavioral Style Scale indicate greater tendencies for monitoring information-seeking preference, rather than blunting information-seeking preference.

##### Self-help Compliance

The Self-Help Compliance Scale [47] is a brief measure assessing engagement with self-guided psychological interventions. The scale consists of 3 items presented on a 5-point Likert-type scale assessing the amount of information participants read (anchored from *0%* to *100%*), the number of suggestions and worksheets participants completed (anchored from *0%* to *100%*), and how much time participants spent using the program per week (anchored from *None* to *61+ minutes*). The questionnaire also includes one open question asking participants what other psychological treatment they had received during the program.

We also predict that psychological flexibility will mediate the effect of the UK-adapted FMW intervention. We operationalized psychological flexibility using CompACT [48].

##### Psychological Flexibility

The CompACT [48] is a 23-item self-report measure of psychological flexibility, allowing the calculation of subscale scores for (1) openness to experience, (2) behavioral awareness, and (3) valued action. Each item is presented on a 7-point Likert scale ranging from 0 (*strongly disagree*) to 6 (*strongly agree*). The CompACT has adequate internal consistency reliability (average interitem correlation, *r*=0.34), good convergent validity, and good discriminant validity [48]. Higher scores on CompACT indicate greater psychological flexibility.

#### Embedded Qualitative Interviews

We will purposively recruit 20-30 participants from the intervention group (ensuring a range of age, gender, cancer type, and website engagement) to participate in a semistructured interview 2-4 weeks after trial completion. Semistructured interviews will be used to allow flexibility in the focus of interviews for each participant [49], in-depth probing of individuals’ experiences using the FMW UK website, and factors that affect acceptability and engagement. Participants willing to take part in this embedded study will be offered the option to complete the interview in person (either at the university or in their own home) or via telephone or video call, provided the chosen interview mode adheres to any government and workplace COVID-19-related social distancing rules at the time. Any travel cost will be reimbursed. Our interview topic guide will probe for participants’ frequency of website use and, if applicable, reasons for low use, overall evaluation and perceived usefulness of the FMW program, and any suggestions for improvement, which are important components of acceptability and will be used to inform both refinements of the intervention materials and any planning for implementation after the trial is complete. All qualitative interviews will be audio-recorded and transcribed verbatim for later analysis.

### Analysis

The analysis plan matches the Australian FMW RCT [27] as closely as possible. Members of the research team involved in the analysis will be blinded to the condition allocation until the end of the trial. First, we will conduct data cleaning to ensure that all data values are possible and plausible. Errant data entries will be deleted from the final analysis data set and missing data will be handled using either prorating or imputation methods as is (1) appropriate to the collected data and (2) congruent with the specific scoring instructions for the psychometric measure from which there is a missing response.

Descriptive statistics will be used to provide sample characteristic information and to identify any potentially prognostic demographic or clinical covariates. Inferential statistical analyses are powered to undertake mixed model repeated measures analyses to examine intervention effects on change from baseline to follow-up for each outcome, using intention-to-treat analysis. Two models will be run for each: (1) unadjusted, accounting for covariance of baseline measures of outcomes and (2) fully adjusted, controlling for all potential confounding variables assessed. Where possible and adequately powered, we will include potential confounders in our analyses and evaluate the effects of missing data using sensitivity analysis. Cohen *d* effect sizes reflect intervention effects, and clinically significant changes will be assessed using reliable change indices. The health care use outcome will be summarized descriptively for activity counts and cumulative costs estimated by assigning unit costs to units of activity. Cost summaries are derived from discrete payer perspectives. Generalized linear models will be used to adjust for the same confounding variables as in the efficacy analysis. All quantitative data analyses will be undertaken in R software (R Foundation for Statistical Computing) [50] where possible, with any supplementary analyses conducted in IBM SPSS as appropriate*.*

Qualitative data collected during the embedded qualitative interviews will be analyzed using thematic analysis [51]. In accordance with best practice guidance for thematic analysis, analysis will be undertaken by one member of the local research team with a proportion audited independently by a second researcher. A small subgroup of the trial steering group will then be convened to review the preliminary thematic structure and provide feedback. Qualitative analysis will be performed using the NVivo software (QSR International).

### Data Sharing Plan

As part of our commitment to transparent open science practices, anonymized quantitative data sets generated from the trial will be stored and made available through the Open Science Framework following the publication of trial findings. These data will include the primary and secondary outcome measures, demographic and clinical data, and any moderating or mediating variables that we ultimately include in all planned and exploratory analyses. We will not include the name of the participants’ recruiting cancer centers in the interest of maintaining participant anonymity. Participants will be asked to explicitly consent for their anonymized data to be shared with other members of the research community in this way.

Given the focused nature of the qualitative interview schedule (ie, engagement with, views on, and suggestions for improving FMW UK), and the ethical risks involved in releasing qualitative data openly because of the difficulties in adequately deidentifying data, we do not currently plan to share data from this aspect of the trial. However, we will review best practice guidelines as they change over the course of the project and review this aspect of the data sharing policy at the time of project completion.

### Monitoring of Adverse Events

We have risk-assessed the potential for serious adverse events from this clinical trial to be low. When a member of the research team is contacted by a participant reporting an adverse event (including elevated psychological distress), they will follow a standard protocol to assess the seriousness of the situation. In the case of disclosure of suicidality and immediate safety concerns, the researcher will contact emergency services and remain on the telephone with the participant until they arrive. In all other cases, the researcher will provide signposting to additional psychological support available as part of standard care, including to the general practitioner and clinical team. All adverse events will be reported to the principal investigator who will assess the severity of the event and report it to the study sponsor (and NHS Research Ethics Committee in the case of a serious adverse event). Provided that participants have provided consent for us to do so, we will also report the adverse event to the clinical team so that a member of the relevant care team can contact the participant to ensure that appropriate support is put into place.

### Ethical Approval and Trial Registration

Ethical review was sought from the University of Chester Department of Psychology Ethics Committee to trigger agreement from the university to act as study sponsor. Full approval was obtained from the NHS Research Ethics Committee (reference: 21/WA/0029), leading to the approval of the Health Research Authority, followed by site-specific research governance approvals at each site. As one of our sites is in Wales, professional Welsh translations of study information are being provided for use at that site, in accordance with the Welsh Language Act (1993) [52]. The trial was registered on the ISRCTN (International Standard Randomized Controlled Trial Number; reference: ISRCTN14317248; date registered 08/04/2021). We have established a trial profile on the Open Science Framework (DOI 10.17605/OSF.IO/ZSHBQ; date registered: May 18, 2021) to facilitate the later sharing of data. The trial was designed in accordance with the principles for medical research involving human subjects, as laid down in the World Medical Association Declaration of Helsinki.

## Results

The grant for this trial was awarded by the North West Cancer Research in September 2019. The project commenced in April 2020, but the initial progress was slower than expected because of the impact of the COVID-19 pandemic on health research in the United Kingdom [53].

### User Testing of the Adapted FMW Program

All intervention adaptation work was completed by January 2021. User feedback from our panel of three health care professionals and four cancer survivor volunteers was then collated. The overall response was positive, with health care professionals noting that the program was helpful and supportive and that they would recommend it to their patients. The cancer survivors also praised the program, stating that they wished they had had access to something similar during treatment. Some minor changes were recommended, as summarized below.

First, a number of technical issues were highlighted and corrected, including the following:

- The website tutorial and resources tab were made to be more prominently visible through altered placement and graphical appearance on the webpage.
- Some navigational issues were also highlighted, with some links not working and others navigating to the wrong page.
- The embedded YouTube videos were set up as playlists, which means that each one, on completion, linked to the next video in the playlist, giving a preview to what was to come in other modules. YouTube has the option to easily disable this feature.
- The linked content was reprogrammed to launch in a new tab to prevent users from becoming lost in the underlying web architecture.

Second, user feedback highlighted some areas where content could be more inclusive. For example, some occurrences of gendered language were replaced with more inclusive language (they or them), and the skin tone of some cartoon images was varied to represent the population diversity of our target recruitment area. Minor changes were made to correct a perceived bias toward breast cancer and to be more inclusive of those without a faith belief or religion.

Third, some aspects of terminology were perceived as outdated (eg, *taking the telephone off the hook*) and were thus replaced (eg, *turning your mobile off*). Similarly, recommendations for meeting new people and maintaining social support were updated to reflect the drive toward social media over traditional media. Some minor changes were made to the language used to refer to different types of health care professionals used in the UK health care system.

Finally, changes were recommended to the flow of the *Improving Communication* page and a greater range of linked or recommended charities and support organizations were added to the support pages. One participant recommended changes to the *Sexuality and Intimacy* section related to safe sex practices during cancer treatment, which were then researched and rewritten by our team to align with current NHS guidance [54].

### Clinical Trial Progress

We launched recruitment for the clinical trial in late April 2021, initially at our two largest hospital centers. The remaining sites will begin recruiting from the summer of 2021. We plan to complete recruitment by February 2022, with all follow-up quantitative data collection completed by October 2022 and all qualitative interviews completed by December 2022. Data analysis will then take place. We aim to report on the findings from the trial from spring 2023.

## Discussion

### Trial Status

The FMW program of psychological support has yielded promising results among recently diagnosed adult cancer survivors in Australia [25-27]. However, the adaptation work described in this protocol was necessary to make this program suitable for implementation in the United Kingdom. Including equipment, web design, videography, and patient and public reimbursement, our adaptation work has costed in the region of £25,000 (US $34,593; excluding staffing costs), taking approximately 10 months to complete. This was a considerable undertaking but still represents a very substantial cost saving compared with developing a new intervention from scratch [55,56]. These efforts were important and justified, given the positive feedback reported by our user testing group. Importantly, our approach to adaptation of the website content allowed us to adopt some elements of co-design with patient experts [57], as recommended by the UK NIHR [58]. This approach to close—and *active*—partnership work with our broader expert stakeholders will not only increase the acceptability of our adaptation [59] but will also enhance the possibility for later implementation and impact [60] across the United Kingdom, should this trial demonstrate efficacy.

The FMW UK clinical trial, which is now underway, will test the efficacy of this program in reducing cancer-specific distress, improving well-being, and reducing the need for broader health care use. As much as possible, we retained (or improved) features of the original Australian RCT to ensure that our work can act as a replication trial. Replication studies are an important aspect of the scientific process [61] and have an important place in psychological [62], broader health sciences [63], and clinical trials [64] literature. Our mixed methods design is important to the integrity of our trial and offers not only efficacy and cost-effectiveness information but also information on the sociocultural context and lived experiences of participants engaged in the intervention [65].

If our UK-based trial does indeed replicate the Australian findings, then this research study will have identified a novel and cost-effective method of psychosocial care delivery for cancer survivors in the NHS. This will of course be limited to those particular sites from which we are recruiting (ie, in North West England and North Wales), and so some additional work may need to be undertaken to explore potential barriers and appropriate pathways for rapid implementation and evaluation across other parts of the United Kingdom.

### Dissemination Plans

To contribute to the transparency of our clinical trial, a full and detailed trial protocol is available as an open resource through the Open Science Framework (DOI 10.17605/OSF.IO/ZSHBQ).

Our primary scientific dissemination will be through high-quality peer-reviewed journal articles and relevant national and international conferences. We will prioritize journals and conferences that maximize dissemination to cancer care clinicians as well as psychosocial oncology researchers. Our study is registered on the NIHR CRN Cancer Portfolio, and we will work with the NIHR, NHS sites involved in recruitment, and with our existing network of charity partners to maximize dissemination opportunities. We will ensure dissemination to the public through regular newsletters to trial participants and an annual public lecture event. Our cancer survivor coinvestigator will be part of the authorship team for all of our dissemination activities, and we aim to include our additional patient, caregiver, and health care stakeholders on the trial steering group in contributing to lay summaries and public dissemination activities.

On completion of the FMW UK trial, our dedicated *YouTube* channel containing both health care professionals and edited cancer survivor videos will be publicly listed to ensure maximized societal benefit.

## Appendix

Multimedia Appendix 1 Peer-reviewer report from North West Cancer Research (UK).

## Abbreviations

CRN: Clinical Research Network
FMW: Finding My Way
ISRCTN: International Standard Randomized Controlled Trial Number
MOS: Medical Outcome Study
NHS: National Health Service
NIHR: National Institute for Health Research
QLQ-C30: European Organisation for Research and Treatment of Cancer Quality of Life Core Questionnaire
RCT: randomized controlled trial
RN: research nurse
