# Supplementary material for: The Finding My Way UK Clinical Trial: Adaptation Report and Protocol for a Replication Randomized Controlled Efficacy Trial of a Web-Based Psychological Program to Support Cancer Survivors
Source: JMIR Res Protoc. 2021 Sep 20;10(9):e31976. doi: 10.2196/31976 (PMC8491121; doi:10.2196/31976)
Supplement: Multimedia Appendix 1 [file resprot_v10i9e31976_app1.pdf]

north west cancer research
Putting our region's cancer needs first

Re: Project Proposal AR2019.08
Title: Finding My Way UK: Adaptation and replication testing of the benefits of online psychological support for cancer survivors.

Investigators: Prof Nicholas Hulbert-Williams, Dr Lee Hulbert- Williams, Dr Lisa Beatty, Prof Bogda Koczwara, Dr Laura Ashley, Prof Neil Coulson, Dr Peter Hall, Prof Eila Watson, Mrs Sue Millington, Mr Richard Jackson

Reviewer feedback:

Reviewer 1

1. THE APPLICANTS

a) How familiar are you with the work of the applicant(s)

? With the lead applicant yes.

b) Have you ever collaborated with and/or published with the applicants?

NO.

c) How do you rate the research standing and ability of the applicants to carry out the proposed work? Very good.

2. IMPORTANCE

How important to the understanding of cancer are the aims of the proposed work? In what way are they important?

This proposal presents important and significant potential to advance the psychological understanding and the capacity to provide good evidence-based care to a population neglected. I like the communication plan - for dissemination and public engagement. This has the potential to help many thousands of cancer patients - it is innovative and forward looking. The research team behind it appear to be of excellent caliber - demonstrated by previous peer reviewed publication & a commitment to this area of research.

3. ORIGINALITY

Have others attempted to answer the same questions (or are they doing so now)? If so, please elaborate and indicate whether the work needs repeating or if the proposal offers anything new. This proposal seeks to test the suitability of a programme for a particular population. it is therefore unique, novel and potentially of great value.

4. METHODOLOGY Is the Plan of Investigation likely to yield important results and, if so, on what time scale? Please give reasons and feel free to suggest improvement. The methodology strikes me as very well considered and very appropriate to the topic under investigation.

. LEVEL OF FINANCIAL SUPPORT Please comment on the costs requested for:

(a) Staff - appropriate

(b) Expenses – appropriate

(c) Equipment

All appropriate

6. CONCLUSION This is a very well considered and well articulated proposal. it tapps into an area that requires research that I understand the team applying can deliver on. The proposed time frame seems very realistic. I believe this is a very important piece of research and commend the application.

Reviewer 2

1. THE APPLICANTS

   a) How familiar are you with the work of the applicant(s)?

      If Yes, please state the dates of collaboration and/or publications within the last five years.

I am aware of the applicants' work in the field.

[redacted]

   b) How do you rate the research standing and ability of the applicants to carry out the proposed work?

They applicants have produced high quality outputs, ensuring a rigorous approach to their research.

2. IMPORTANCE

   How important to the understanding of cancer are the aims of the proposed work? In what way are they important?

The aims of the proposed work are important, as distress and anxiety are common during treatment for cancer, and effective evidence-based programmes are lacking. However, I question whether the proposed programme would be of greater benefit post-six months following diagnosis/treatment. This is often the time (once active treatment has ceased) when survivors are particularly struggling. I wonder whether this may also be why effective findings were not found in more outcomes in the Beatty et al paper (although I also appreciate this may be due to the nature of the control condition). Nonetheless, helping relieve psychological distress among survivors is of grave importance.

3. ORIGINALITY

   Have others attempted to answer the same questions (or are they doing so now)? If so, please elaborate and indicate whether the work needs repeating or if the proposal offers anything new.

Obviously, this trial is a replication of a previous study; therefore, it is not necessarily original. However, it involves the adaptation of the programme. Based on the findings from the previous study, the programme warrants a larger RCT, as the programme has been developed in a rigorous evidence-based manner, and indicated promising effect sizes. However, whilst it is not an original programme, it would require adaptation for the UK context, and could offer benefits to the cancer survivors accessing the NHS.

4. METHODOLOGY

   Is the Plan of Investigation likely to yield important results and, if so, on what time scale? Please give reasons and feel free to suggest improvement.

The proposed methodology has been well thought out, and adopts a rigorous approach – ensuring that the adaptation/development work is not rushed. It is likely to yield important results, in terms of whether the programme is effective, by the end of the 2.5 years. What has not been accounted for in the timeline, and should be, is time to implement further changes to the programme based on the qualitative findings. This is important, particularly if the programme is to be rolled out.

A concern of mine also relates to evaluating the programme among all cancers, regardless of the diagnosis. Whilst there will be commonalities of experiences across the different cancer groups, there is likely to be concerns specific to the cancer itself which will not be addressed, or are irrelevant to some. The prior Beatty et al evaluative study was primarily made up of breast cancer patients, and therefore is not necessarily representative of all other cancers. The more generalised nature of the material (i.e. not specific to cancers) may also be another reason why effective findings were not identified across more outcome measures. It is therefore difficult to know whether the

larger sample size proposed in the present study would overcome this problem, and lead to significant findings.

5. LEVEL OF FINANCIAL SUPPORT

Please comment on the costs requested for:

(a) Staff

This seems reasonable.

(b) Expenses

These seem to have been reasonably calculated.

(c) Equipment

This seems reasonable for the cost of two computers/laptops.

Reviewer 3

6. THE APPLICANTS

c) How familiar are you with the work of the applicant(s)?

I am familiar with the work of the principle investigator and some of the co-investigators via conference presentations and publications in various Health Psychology and Oncology journals.

If Yes, please state the dates of collaboration and/or publications within the last five years.

[redacted]

d) How do you rate the research standing and ability of the applicants to carry out the proposed work?

The research team that has been assembled is excellent. A range of disciplines are represented including health psychology, oncology, health economics and medical statistics. Within this, the research team demonstrates considerable experience of online psychosocial interventions which will be invaluable for the current study. The international collaborative

nature of the team which includes the authors of the original Australian intervention is also a benefit.

The publication records of the research team demonstrate an ability to publish high quality papers related to psycho-oncology. Members have also secured funding for complementary studies, although in some cases these are small grants.

7. IMPORTANCE

   How important to the understanding of cancer are the aims of the proposed work? In what way are they important?

As outlined in the study, distress among cancer survivors remains burdensome both to the individual in terms of quality of life and to the health service in terms of increased costs. There is currently little available to support psychosocial wellbeing of people diagnosed with cancer. Although not addressed in this study, distress is likely to also be related to adherence to adjuvant medication and lifestyle modifications that may be required to reduce recurrence after the primary cancer is treated. A number of recently published studies have identified that depression and anxiety are important issues among cancer survivors and often constitute an unmet need, particularly as patients are discharged into open access follow-up where this type of non-clinical outcome is difficult to monitor and address by the clinical nurse specialists. An online intervention may therefore be particularly feasible and desirable for this population.

8. ORIGINALITY

   Have others attempted to answer the same questions (or are they doing so now)? If so, please elaborate and indicate whether the work needs repeating or if the proposal offers anything new.

There is a lot of research currently being undertaken in the area of e-health in psycho-oncology. However, this application is noteworthy because of the efficient design of testing an intervention which has already been developed in a different country. This will provide both important replication data and provide the opportunity to tailor the intervention to be culturally specific to a different healthcare system. As the original trial showed improvements in quality of life and distress over time but no group differences, this study would be improved by including both an active control (in line with the previous study) and TAU in order to determine true intervention effects. This would be beneficial not only for the local implementation of the intervention but also for the global perspective.

9. METHODOLOGY

   Is the Plan of Investigation likely to yield important results and, if so, on what time scale? Please give reasons and feel free to suggest improvement.

Overall, the methodology plan is suitable to trial this type of online psychosocial intervention. The timescale is mostly appropriate, although if ethical approval is required from the institution before sponsorship can be agreed for the NHS HRA ethics application, then this may require some additional time – time for amendments to add additional recruitment sites if necessary

should also be considered as this can take up to 3 months. The costs and time that have been allocated to modify an existing intervention rather than design a new one are realistic and represent good value for money.

There are a number of positive highlights from the proposed study. The research team have designed a high quality replication of a previous study which showed promising results in Australia. Replication is an important and often overlooked part of the scientific process. A good recruitment strategy has been proposed and the researchers have evidenced that they have worked with the sites successfully in previous studies. As the study would be eligible to be listed on the CLRN portfolio and the recruitment process is not too onerous on the research nurses, this should be a successful strategy. The researchers have also embedded high quality PPI into the study from writing the application through to dissemination with an expert patient co-applicant.

There are some comments on the methodology that could be addressed;

1) The study on which this trial is based showed no significant differences between groups on the primary or secondary outcomes, which the authors postulate is because of the active control. The treatment as usual (TAU) group proposed for the current study appears to not be a true TAU and may weaken the effect. Given the results of the previous study in Australia, it would be useful to employ a three arm trial of intervention, active control and TAU to determine, although that would require a larger sample size.
2) As all types of cancer are eligible for the study, this may dilute the effect of the intervention. The content could be more specific and tailored to particular cancer-specific issues which may increase the effectiveness. The researchers state that "efforts will be made to balance cancer types between groups" but it is unclear how this will be done. Will stratification by cancer type be carried out?
3) Participants will not be screened for distress. Although I understand that the researchers intend to replicate a naturalistic setting, the lack of screening will minimise the potential effectiveness of the intervention; i.e. if a participant is not distressed then it is not possible to improve this outcome. Although an online intervention, resources would be required to implement the intervention into usual care and therefore it may be more prudent to concentrate on those who most require it. Alternatively, the sample size calculation should take into account a suitable sensitivity analysis to determine the effects for those most distressed, which does not currently appear to be the case.
4) There is currently little information about how distress and series adverse events will be detected and how the routes for referral. Will content of the website be monitored, and if not, will this be made clear to participants. These issues however will be covered as part of the ethics application.
5) Emotional factors are well covered in the measurement of outcomes, however cognitive factors (e.g. illness perceptions) are not. These are likely to be related to some of the outcomes of interest and process variables.
6) Recruitment options are sensible, however the researchers may consider including more funds to cover advertisement for online recruitment and postal data collection.

10. LEVEL OF FINANCIAL SUPPORT

Please comment on the costs requested for:

(d) Staff
(e) Expenses
(f) Equipment

Costs are mostly appropriate, however the researchers may consider including more funds to cover advertisement for online recruitment and postal data collection.

It would be appropriate for the PI and principle researcher to visit the intervention development team in Australia and the costs included for this are justifiable. However, the backfill for the one week of PI team could be reconsidered.

Panel reviews

1. The applicants state that over one-third of cancer patients report anxiety or depression. The North West, as everyone here will know, has a higher rate of cancer diagnosis and deaths compared to the rest of the UK.

2. Finding my way is originally an Australian Web-based modular programme developed by two of the team submitting this proposal, that is Dr. Beatty a d Bagda Kaswara.

3. Indeed, Dr Beatty has already conducted a RCT on the programme in Australia which demonstrated reduced distress and healthcare utilisation amongst the cohort using the application.

4. However, this project needs to make this six-part modular programme "UK friendly". There needs to be a re-production of both patient and clinical staff interviews to produce videos which are culturally relevant to the UK.

5. The applicants state that patients will be eligible if they have been diagnosed with cancer in the last six months and received anti-cancer treatment with a curative content.

6. It is proposed that 294 patients will be placed into 2 groups. Half will be given access to the newly formatted programme. The other half will have access to standard care.

7. We are told that those with access to standard care only, will in fact be able to access the programme at the end of the study.

There has been involvement of a cancer survivor in the application as a co-investigator, and the team will use survivors and clinical staff in the "UK remake" of the interviews for the programme. There will also be cancer survivor involvement on the steering group.

AIMS

1. The aims are to primarily adapt the Finding My Way programme to the UK audience.
2. It will replicate the Australian findings of reducing stress in those diagnosed with cancer.

3. If the Australian outcomes are replicated and stress is reduced, this in turn may reduce healthcare utilisation.

STRENGHTS

1. Finding My Way, as an intervention, has already been subjected to rigorous review.

2. The team consists of the originators of the intervention and a mix of disciplines, all with many publications on the psychological aspects of oncology.

3. Success has previously been demonstrated.

COMMENTS

1. Although the methodology has presented his tried and tested, one reviewer suggests this could be improved by the introduction of a third group - an active control. Thus giving:
    a. the intervention group
    b. TAU group
    c. The active control group

2. the study covers all types of cancer currently, but that means the sample for each type will below. With the program work better for particular types of cancer?

3. Mixed feelings amongst viewers about the costs for travel to Australia. Whilst it may be desirable, I don't feel that this is essential. Work can be shared and discussed via IT solutions.

4. I don't feel that this is so special and application to ask NWCR to deviate from guidelines of not paying back-fill costs.

Strictly speaking this work is based on the Australian original model and has already been rolled out in the United States and Romania.

However, the overall aim of the original team should this be successful, is to give Open Access to a YouTube version. Therefore, I would see this as contributing significantly to the advancement of psycho-social management of cancer in the international environment

Panel review

This is a high quality replication study that addresses an important national need to develop and evaluate evidence-based supportive interventions for cancer survivors in the UK; it also addresses local need, with patients in the North West more likely to be affected by cancer. Online psychosocial interventions offer the potential for low-cost intervention with scale/reach. The applicants propose to adapt and trial “Finding My Way” a six week online support programme designed to help patients cope with cancer treatment, originally developed and trailed in an Australian context. Peer-reviewed published data demonstrates promising effects, with reduced distress and healthcare usage in Australian cancer survivors. Who received the intervention. While the intervention isn’t novel, the proposed study is efficient because the applicants can adapt the intervention context and trial design to the UK NHS context. If the trial is successful the findings could have high impact for UK NHS cancer services

A strong team that includes expertise/track record in psycho-social oncology and methods including online health interventions, health economics and trails. That’s the original intervention developers are involved as co-applicants is advantageous and will support international collaboration and capacity building. The PPI plan is high quality, with involvement of PPI at co-applicant level and a sound plan for involving additional people who have been affected by cancer in the study steering group.

The methods are robust and presented in detail, including randomised control trial with nested qualitative interviews and quantitative follow up at 3 and 6 months. A suitable sample size calculation is provided. The timeline seems feasible. Analysis plans are given in sufficient detail. Costs are justified in considerable detail; the

total £297,630 is good value for money for a well-designed trial of an existing (adapted) intervention

Major comments

Rev 1 & 2 both expressed concern that by including all cancers the intervention effect may be diluted due to not addressing concerns and experiences that are specific to some cancers (although I think the Beatty trial also included patients with different tumour sites). The team does intend to adapt the programme for the UK NHS context, including videos with patients and health care staff for the intervention website, so there may be an opportunity to address this issue. I note that the applicants could build in some time for testing the intervention adaptations for acceptability e.g. the video snippets could be piloted with patient representatives before hosting them on websites.

Minor comments

The applicants are transparent in noting that the recent Australian trial (Beatty et al; 2019) reported improvements in both the intervention and control arm, with no between-group differences. They speculate that this could reflect design aspects such as the control arm (an online task) being too similar to the intervention. I think this underlines the importance of testing what looks to be a promising intervention in a different context and with a larger and fully powered RCT, and the applicants have taken the opportunity to build in a usual care/wait-list control rather than an active control arm
The plan for qualitative process interviews could be fleshed out e.g. what's the rationale for 18-20 qualitative interviews (data saturation)? Can the interview topics be expanded to include barriers/enablers to implementation, which would then inform a future phase of intervention scale-up and roll out?

north west
cancer research
Putting our region's cancer needs first
